# Knowledge, attitudes, practices of occupational accident prevention and related factors among rice farmers in Northern Vietnam

**DOI:** 10.1371/journal.pone.0328474

**Published:** 2025-07-15

**Authors:** My Ha Nguyen, Toan Van Ngo, Anh Quynh Tran, Linh Gia Vu, Nga Thu Phan, Son Truong Pham, Bach Xuan Pham, Tu Duc Pham, Hai Minh Vu

**Affiliations:** 1 Department of Health Organization and Management, Faculty of Public Health, Thai Binh University of Medicine and Pharmacy, Thai Binh, Vietnam; 2 Institute for Preventive Medicine and Public Health, Hanoi Medical University, Hanoi, Vietnam; 3 Thai Binh Medical College, Thai Binh, Vietnam; 4 Department of Trauma, Thai Binh University of Medicine and Pharmacy, Thai Binh, Vietnam; Canadian University Dubai, UNITED ARAB EMIRATES

## Abstract

**Background:**

This study aimed to assess rice farmers’ knowledge, attitudes, and practices (KAP) regarding the prevention of occupational accidents, along with identifying related factors.

**Methods:**

An analytic cross-sectional study was conducted in six rural communes in Thai Binh province, targeting rice farmers aged 18 and older. A structured KAP questionnaire on occupational accident prevention was developed and pilot-tested with 168 participants. Knowledge scores ranged from 0–20 (1 point per correct answer), attitudes from 9–45 (5-point Likert scale), and practices from 17–85 (5-point frequency scale). Higher scores indicated better outcomes. Tobit regression was used to examine factors influencing KAP, accounting for the bounded nature of these scores.

**Results:**

Among the 1,171 rice farmers surveyed, 17.5% had experienced an occupational accident in the six months prior to the study, most commonly involving cuts from sharp tools, falls during fieldwork, or injuries from handling machinery. The average knowledge score on accident prevention was 13.82 ± 4.58 points. The average attitude score regarding accident prevention was 34.47 ± 4.73 points, with the highest scores reflecting a strong emphasis on personal safety in rice farming. Conversely, the lowest scores were related to the perceived importance of seeking immediate medical aid after an accident. The average score for practices related to accident prevention was 63.63 ± 2.22 points. The study found that better KAPwere associated with a lower incidence of occupational accidents.

**Conclusion:**

This study highlights the need for comprehensive health education programs to enhance rice farmers’ KAP in preventing occupational accidents, thereby reducing the risk and impact of such incidents in this population.

## Introduction

An occupational accident is an unforeseen and undesirable event that results in injury to individuals, damage to property, or disruption of labor processes [1]. Occupational safety and health is the scientific discipline that focuses on predicting, identifying, evaluating, and mitigating workplace hazards that could compromise employees’ health and well-being, while also potentially impacting the environment [2,3]. Among various occupational groups, farmers are particularly vulnerable to numerous work-related risks and hazards that can adversely affect their health.

Rice farming presents a unique set of occupational risks due to the nature of its physical demands and the increasing use of machinery and chemicals. The methods rice farmers employ today differ from traditional techniques, as technology has enabled machines to replace human and animal labor in many farming tasks. However, this technological shift has also led to workplace accidents. Sprains and strains account for approximately one-third of injuries that prevent rice farmers from working, while back injuries constitute about one-quarter of such cases [4]. Common types of accidents among rice farmers include superficial injuries and open wounds from sharp objects (62.74%), physical trauma from falls or accidents involving motor vehicles or tractors (18.49%), and acute poisoning from pesticide exposure (8.11%) [5]. These occupational accidents place a significant financial and health burden on farmers, negatively affecting their productivity and quality of life [6].

Improper attitudes toward labor safety and hygiene, along with insufficient knowledge, further contribute to unsafe work practices, leading to a higher risk of accidents [7–11]. Studies show that farmers who lack guidance or equipment for using personal protective gear at work are more prone to accidents, injuries, and occupational diseases than those who are better equipped [12]. For example, a systematic review by Sapbamrer and Thammachai emphasized that education level, awareness, access to training programs, and behavioral norms significantly affect personal protective equipment (PPE) use and pesticide safety practices [13]. Likewise, Keifer highlighted that while PPE and safety procedures can reduce pesticide exposure under controlled settings, their implementation in real-world agricultural environments remains inconsistent and often ineffective without proper interventions [14].

Efforts to improve farmers’ understanding of pesticide safety, such as the use of pictograms and color-coded labels, have shown mixed results. A recent study in Lebanon by Abou Ibrahim and colleagues reported that although a majority of farmers had moderate understanding of pesticide labels, there remained a weak correlation between comprehension and actual safety practices [15]. The limited effectiveness of such labels—especially among older or less-educated farmers—suggests the need for tailored interventions and training. In support of this, Maddah et al. found that a brief educational intervention significantly improved Lebanese farmers’ knowledge, attitudes, and practices regarding pesticide safety [16]. Nevertheless, Dugger-Webster and LePrevost argue that user comprehension of pesticide labels continues to be a global challenge, with users in both developing and developed countries often misunderstanding critical safety instructions [17]. Therefore, increasing awareness of occupational hazards and safety is essential, along with regularly assessing farmers’ knowledge, attitudes, and behaviors related to workplace safety [18].

Rice farming has long been the primary occupation for Vietnamese farmers, and this remains true today. In a recent study, 50.9% of rice farmers in Thua Thien Hue, Vietnam, reported experiencing an accident. Sharp objects were responsible for 58.8% of all accidents, followed by falls at 29.9%. The most common types of injuries were scratches (58.4%), fractures (16.7%), and sprains (13.6%). Injuries were most often sustained to the shoulders and arms (56.6%), followed by the thighs and calves (32.6%). Additionally, 14.9% of accidents had a significant financial impact on households [19].

In Vietnam, small-scale farmers continue to carry out most agricultural activities manually, placing them at increased risk for occupational accidents and injuries. Despite this, training on occupational safety and health remains limited for farmers nationwide [20], and this issue is particularly pronounced in provinces like Thai Binh, where formal safety education is sparse and largely inaccessible [21,22]. Thai Binh is the second-largest rice-producing province in northern Vietnam, with approximately 140,000 hectares of rice cultivated annually [23]. The province plays a crucial role in national food security and rice export capacity, making the health and productivity of its farmers especially important. However, no studies to date have systematically assessed the KAP of rice farmers in this region regarding occupational safety. The lack of evidence-based understanding of farmers’ safety practices hinders the development of effective policies, educational interventions, and protective regulations. Consequently, preventable injuries and long-term health problems may persist, reducing labor capacity and increasing healthcare burdens. Given the province’s agricultural importance and the evident gap in research and safety interventions, this study was conducted to evaluate the current state of occupational safety KAPamong rice farmers in Thai Binh and to identify factors associated with the prevention of occupational accidents. The findings aim to inform context-specific strategies to improve safety conditions in rice farming communities across Vietnam and other comparable low-resource agricultural settings.

## Methods

### Study design and participants

This analytic cross-sectional study was initiated in February 2024 to examine the KAP related to occupational accident prevention among rice farmers in Thai Binh province, located in northern Vietnam. The study also aimed to identify factors associated with these outcomes to inform future occupational health interventions.

#### Study location profile.

The study was conducted in Thai Binh province, a key agricultural hub in the Red River Delta region of northern Vietnam, with a population of approximately 1.86 million people. The province comprises seven rural districts and one city, all with active agricultural economies. Among these, Vu Thu and Kien Xuong districts were selected using a random sampling method from the list of rural districts. These two districts were chosen due to their high intensity of rice cultivation, accessibility for fieldwork, and representativeness of typical rice farming communities in the province. Within each district, three communes were randomly selected, totaling six communes for the study. These selected areas share similar demographic, economic, and environmental characteristics, making them suitable for investigating occupational health issues in rice farming. The population is predominantly of Kinh ethnicity, with little ethnic variation likely to influence beliefs or practices.

#### Sample size estimation and sampling method.

The sample size was calculated based on the assumption that approximately 50% of farmers would demonstrate adequate occupational safety practices, with a 95% confidence level and a 3% margin of error. A design effect of 1.5 was applied to account for the multi-stage sampling approach, resulting in a minimum required sample size of 1,068 participants. To ensure adequate statistical power and to compensate for potential non-responses or incomplete data, we added an additional 10%, bringing the target sample size to 1,175 farmers. Ultimately, 1,171 rice farmers completed the survey and were included in the final analysis.

A multi-stage random sampling method was used to recruit participants. First, two districts—Vu Thu and Kien Xuong—were randomly selected from the list of rural districts in Thai Binh province. Next, within each selected district, three communes were randomly chosen from a comprehensive list of administrative units, resulting in six communes in total. In each commune, a list of all households was obtained from the local health station, and one household was randomly selected using a computer-generated random number as the starting point. From there, data collectors employed door-to-door recruitment using a systematic approach (e.g., visiting every 3rd household) until the required number of participants was enrolled. This sampling strategy helped ensure representativeness and minimized selection bias.

#### Inclusion, exclusion, and discontinuation criteria.

To be eligible for inclusion, participants were required to be at least 18 years of age, currently reside in one of the selected communes in Thai Binh province, and have been actively engaged in rice farming for at least one year prior to the survey. The lower age limit of 18 was set in accordance with Vietnamese labor laws and ethical research standards, which define adulthood as the minimum age for independent consent and full-time labor engagement. While it is recognized that individuals under 18 may assist in farming, they were excluded to ensure legal compliance and reduce ethical concerns related to interviewing minors.

Exclusion criteria included individuals with cognitive impairments or serious health conditions that would interfere with their ability to complete the questionnaire reliably. Farmers who had permanently transitioned to non-agricultural work within the past year were also excluded, as the focus was on those currently practicing rice farming.

Participants were discontinued from the study if they chose to withdraw at any point during the interview process or if their responses were incomplete due to unexpected interruptions, refusal to answer critical sections of the survey, or signs of distress that warranted ending the interview for ethical reasons.

### Data collection

The data collection team included faculty researchers, healthcare workers from six local commune health stations, and senior-year public health students from Thai Binh University of Medicine and Pharmacy. All interviewers underwent comprehensive training led by a multidisciplinary team of experts, including lecturers in epidemiology, occupational health, and biostatistics from the university. The training sessions focused on standardized interview techniques, ethical considerations, informed consent procedures, and accurate questionnaire administration.

Data were collected through face-to-face interviews conducted at participants’ homes. After providing a brief explanation of the study’s purpose and procedures, each participant was asked to sign a written informed consent form. The interviews were conducted in Vietnamese and lasted approximately 30–40 minutes.

The structured questionnaire used in this study was developed by the research team in collaboration with experts in occupational safety, public health, and social sciences. Its content was informed by an extensive review of global literature and adapted from validated KAP instruments previously used in agricultural health research across Southeast Asia and other low- and middle-income countries [7–11]. In particular, the questionnaire incorporated elements from the WHO’s guidelines on the health implications of pesticide use in agriculture [24] and International Labour Organization (ILO) recommendations for farm safety [25]. These frameworks ensured comprehensive coverage of the KAPrelevant to occupational accident prevention and alignment with international content standards.

To assess its validity and reliability, the draft questionnaire was reviewed by five independent experts—two from occupational health, one from epidemiology, and two with experience in agricultural health promotion—who evaluated the relevance, clarity, and cultural appropriateness of each item. It was then pilot-tested with a sample of 168 rice farmers from a neighboring district in Thai Binh province. This pretesting phase enabled refinement of language, item sequencing, and response options based on participant feedback and expert input.

Psychometric evaluation was conducted using Exploratory Factor Analysis (EFA) to identify the underlying factor structure, followed by Confirmatory Factor Analysis (CFA) to validate the model fit. Goodness-of-fit indicators included a Comparative Fit Index (CFI) > 0.90, Tucker–Lewis Index (TLI) > 0.90, and Root Mean Square Error of Approximation (RMSEA) < 0.08, which confirmed an acceptable model fit. Internal consistency was assessed using Cronbach’s alpha, with values exceeding 0.70 for all subscales, indicating satisfactory reliability. Participants in the pilot study were not included in the final analysis.

The final version of the questionnaire consisted of four main sections: (1) demographic and occupational background, (2) knowledge of occupational accident prevention, (3) attitudes toward safety practices, and (4) self-reported safety practices during rice farming.

### Study tool

#### General information.

This section included data on age, gender (male/female), education level (Equal to or lower than Secondary school/Equal to or higher than high school), household economic status (Self/Wife or husband/Others), years of experience in rice farming, daily working hours in rice farming, and whether the participant had experienced an occupational accident in the last six months (Yes/No/Don’t remember). Participants were grouped into two age categories: < 60 years and ≥ 60 years. This cut-off reflects the standard retirement age in Vietnam and helps distinguish between working-age and older farmers, who may differ in occupational risk awareness and behavior.

#### Knowledge of occupational accident prevention in rice farming.

The original questionnaire included 21 questions assessing knowledge. Exploratory Factor Analysis (EFA) from the pilot study revealed six components, but component 6 contained only one question, which was subsequently removed. The final version of the questionnaire included 20 questions across five categories: 4 questions about preventing accidents from machinery, 3 on preventing animal-related accidents, 5 on physical hazards, 5 on accidents related to plant protection chemicals (PPC), and 3 on ergonomic risks. Each question was scored as either knowing (1) or not knowing (0).

To assess the suitability of the data for factor analysis, the Kaiser-Meyer-Olkin (KMO) score was calculated, yielding a value of 0.862. The KMO score is a statistical measure that indicates the proportion of variance among variables that might be common variance; values closer to 1.0 suggest sampling adequacy for factor analysis. Bartlett’s test of sphericity was also statistically significant (p < 0.001), indicating that the variables were sufficiently correlated to proceed with factor analysis. The overall Cronbach’s alpha for the knowledge section was 0.894, indicating high internal consistency. Confirmatory Factor Analysis (CFA) showed good model fit with a CMIN/df of 1.239.

#### Attitudes toward occupational accident prevention in rice farming.

The attitude component of the questionnaire initially included nine items designed to assess farmers’ perceptions of occupational accident prevention in rice farming. These items were informed by a review of relevant international literature and guidelines on agricultural occupational health and were refined through consultation with experts in public health, occupational safety, and social sciences. The final items were pre-tested during a pilot study with 168 rice farmers to assess clarity, cultural relevance, and reliability. Feedback from the pilot phase led to minor wording adjustments; reverse-coded items were considered but excluded to avoid confusion among participants with lower educational levels.

All nine attitude items were retained in the final version of the questionnaire. Responses were recorded using a five-point Likert scale ranging from 1 (strongly disagree) to 5 (strongly agree), with higher scores indicating more favorable attitudes toward accident prevention. Exploratory Factor Analysis (EFA) revealed a single-factor structure with a KMO value of 0.890 and a statistically significant Bartlett’s test of sphericity (p < 0.001), supporting the appropriateness of the data for factor analysis. The internal consistency of the attitude scale was strong, with a Cronbach’s alpha of 0.833. Confirmatory Factor Analysis (CFA) demonstrated a good model fit, with a CMIN/df of 1.030.

#### Practices in preventing occupational accidents in rice farming.

The initial questionnaire had 19 practice-related items. After EFA, two single-question components were removed, resulting in a final version with 17 questions. These were grouped into four categories: 4 questions on preventing accidents related to PPC, 4 on maintaining health while working, 3 on machinery safety, and 6 on overall occupational safety. Each question was rated on a scale from never (1) to always (5). EFA for the practice section produced a KMO score of 0.830, a statistically significant Bartlett’s test (p < 0.001), and a Cronbach’s alpha of 0.842. CFA confirmed a good model fit with a CMIN/df of 1.495.

Following the statistical testing and cultural adjustments made based on participant feedback, the finalized questionnaire was used for the official survey.

### Statistical analysis

All data were entered and cleaned using EpiData version 3.1, then analyzed using IBM® SPSS® Statistics version 22.0 and STATA version 12. Descriptive statistics were used to summarize participants’ demographic characteristics, agricultural work profiles, and responses to the knowledge, attitude, and practice (KAP) items. Categorical variables were reported as frequencies and percentages, while continuous variables, including KAP scores and related items, were summarized using means and standard deviations (SD).

KAP scores were treated as continuous outcome variables. The **knowledge score** was calculated as the sum of correct responses to 20 binary (yes/no) items across five domains: machinery and equipment, animals, physical impact, plant protection chemicals (PPC), and ergonomics (range: 0–20). The **attitude score** was the total of 9 items scored on a 5-point Likert scale (1 = totally disagree to 5 = totally agree), producing a possible range of 9–45. The **practice score** was derived from 17 items evaluated on a 5-point frequency scale (1 = never to 5 = always), yielding a total score range of 17–85.

To identify factors associated with knowledge, attitude, and practice (KAP) scores, multivariate Tobit regression models were employed. This statistical approach was appropriate because the outcome variables (KAP scores) were continuous but censored, bounded by minimum and maximum possible scores due to the use of Likert-scale-based composite measures. Ordinary least squares regression would not account for this censoring, potentially leading to biased estimates. Separate Tobit models were constructed for each KAP domain to examine the influence of individual-level covariates. Independent variables included gender, age group, education level, years of experience in rice farming, average daily labor hours, engagement in other occupations, and recent history of occupational accidents. The Tobit model allowed for more accurate estimation of associations by correcting for the limited dependent variable range inherent in KAP scoring.

In addition, knowledge and attitude scores were included as covariates in the models assessing attitude and practice, respectively, to explore interrelationships among the KAP components. All variables were included in the multivariable models based on theoretical relevance and findings from bivariate analyses (not shown). Model assumptions were assessed prior to analysis.

All statistical tests were two-tailed, and a **p-value less than 0.05** was considered statistically significant.

### Ethical considerations

The study received approval from the Ethics Committee of Hanoi Medical University (project code: TNLDLUA, approval number: 1054/GCN-HMUIRB). Authorization was also obtained from local community leaders. All participant information was kept confidential and used exclusively for research purposes. Prior to participation, each individual provided informed consent after being fully briefed on the study.

## Result

### Participant characteristics

A total of 1,171 rice farmers participated in the study, with 56.5% being female and 43.5% male. The average age of the farmers was 59.10 ± 12.15 years, and 76.5% had completed high school or less. On average, the farmers had been engaged in rice cultivation for 33.10 ± 13.92 years, working an average of 4.87 ± 2.15 hours per day. Additionally, 17.5% of the farmers reported experiencing at least one occupational accident during the previous six months of rice farming (Table 1).

**Table 1:** Demographic characteristics, agricultural activities and occupational accidents of farmers.

Characteristic		n	%
Gender	Male	509	43.5
	Female	662	56.5
Age group	< 60	487	41.7
	≥60	684	58.3
Education	Equal to or lower than Secondary school	896	76.5
	Equal to or higher than high school	275	23.5
Occupational accident in the last six months		250	17.5
		**mean**	**SD**
Age		59.10	12.15
Number of years working in rice production		33.10	13.92
Number of labour hours in rice production per day		4.87	2.15

### Knowledge

**Fig 1:**
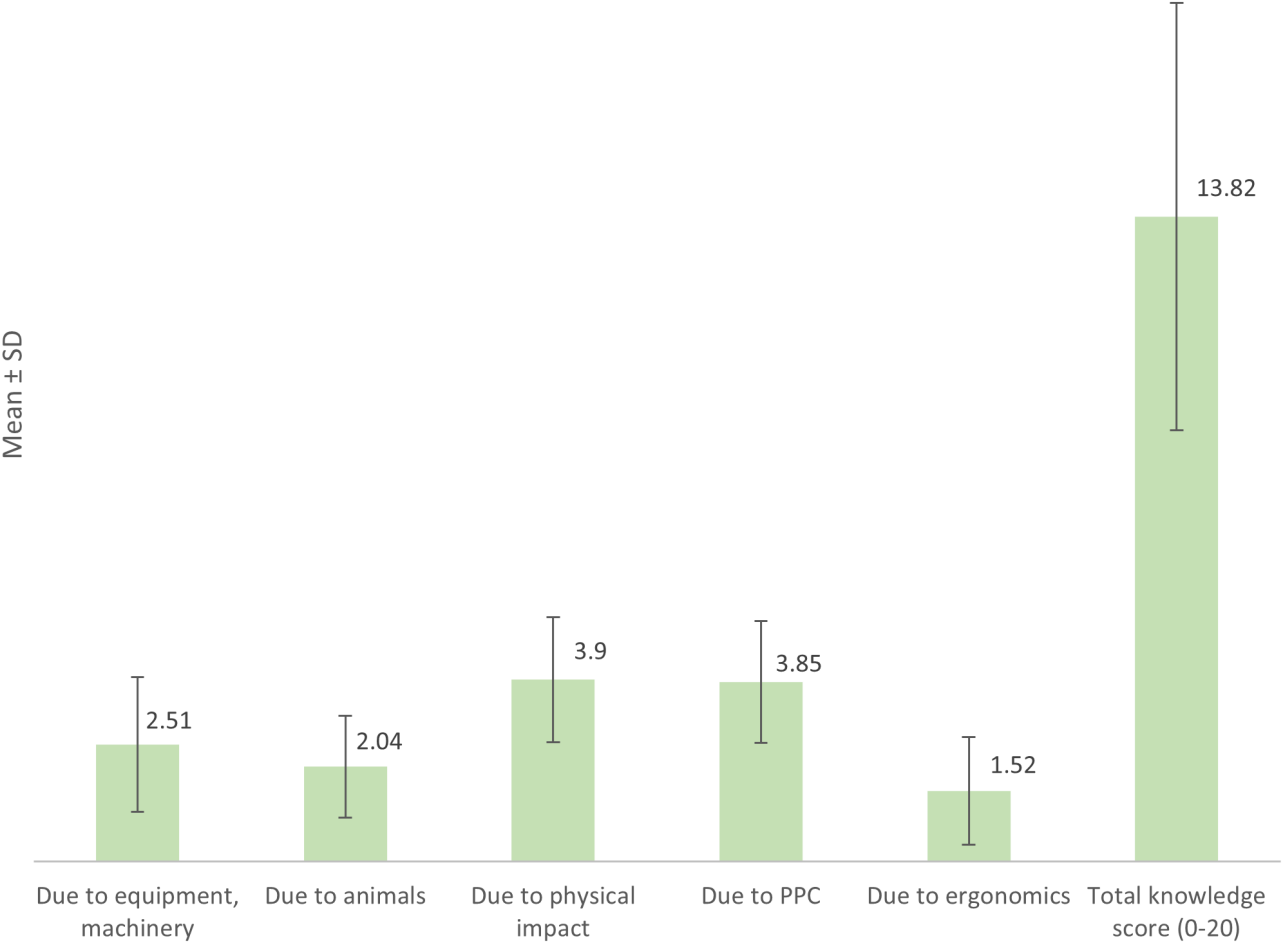
Knowledge of preventing occupational accidents of rice farmers.

The average knowledge score of farmers in preventing accidents related to labor equipment and machinery was 2.51 ± 1.44. For preventing accidents caused by animals, the score was 2.04 ± 1.09, while it was 3.90 ± 1.34 for preventing physical impact-related accidents. The score for preventing accidents due to plant protection chemicals was 3.85 ± 1.30, and for ergonomics-related accidents, it was 1.52 ± 1.15. Overall, the subjects’ average knowledge score was 13.82 ± 4.58 points (Fig 1).

### Attitude

The mean attitude score of rice farmers toward preventing occupational accidents was 34.47 ± 4.73 points. Among the specific attitudes measured, the lowest score was for the need to seek immediate first aid at a medical facility after an occupational accident (3.62 ± 0.77), while the highest score related to the importance of personal safety in rice production (3.93 ± 0.85) (Table 2).

**Table 2:** Attitude of preventing occupational accidents of rice farmers.

Contents	%	%	%	%	%	Mean±SD
	Totally disagree	Disagree	Neutral	Agree	Totally agree	
Occupational accidents in rice production represent a serious concern.	0.3	1.9	27.1	51.7	19.0	3.87 ± 0.74
Every farmer is at risk of experiencing occupational accidents during rice production.	0.3	3.2	24.0	53.5	19.0	3.88 ± 0.76
Ensuring personal safety while working in rice production is of utmost importance.	0.5	2.9	27.8	40.3	28.5	3.93 ± 0.85
Occupational accidents in rice production are preventable.	0.4	3.6	28.8	41.2	26.0	3.89 ± 0.85
Maintaining a safe working environment is essential to minimize the risk of occupational accidents in rice production.	0.3	3.1	28.5	40.3	27.8	3.92 ± 0.84
I am willing to take proactive steps to prevent accidents in rice production.	0.4	2.1	28.6	47.3	21.6	3.88 ± 0.78
Training programs to enhance farmers’ knowledge and practices on occupational safety and hygiene are essential.	0.5	2.4	36.2	43.5	17.4	3.75 ± 0.79
Seeking immediate first aid at a medical facility after an occupational accident is necessary.	0.4	2.8	44.7	38.7	13.4	3.62 ± 0.77
I am willing to remind and encourage other farmers to follow proper safety measures in rice production.	0.3	3.4	35.5	44.2	16.6	3.73 ± 0.79
**Total attitude score (9–45)**	34.47 ± 4.73					

### Practices

**Fig 2:**
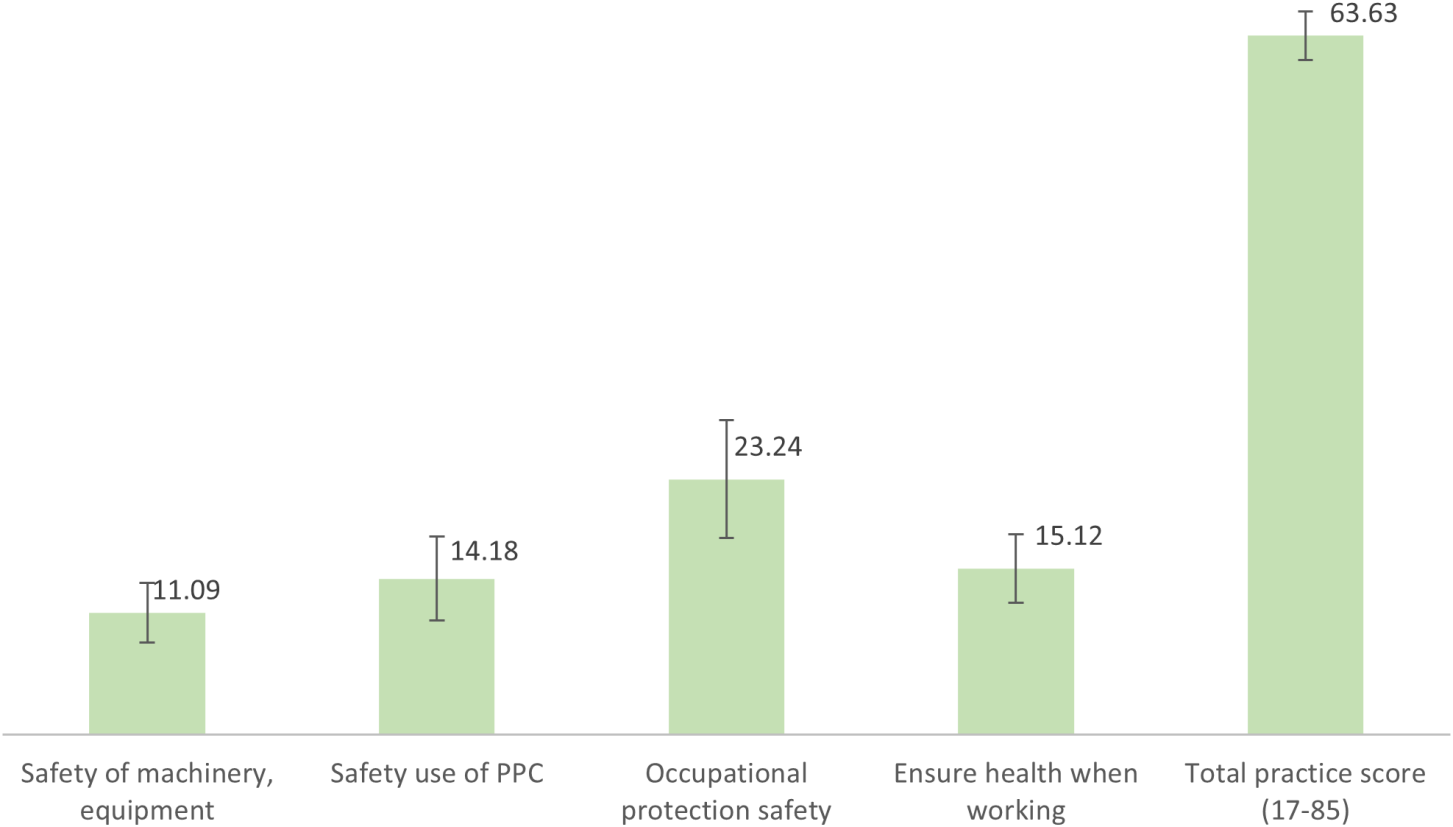
Practice of preventing occupational accidents of rice farmers.

Fig 2 shows that, for the practice of preventing occupational accidents, the farmers had an average score of 63.63 ± 2.22 points. In different areas of practice, the average score for machinery and equipment safety was 11.09 ± 2.70, for safe use of plant protection chemicals (PPC) it was 14.18 ± 3.83, for labor protection safety it was 23.24 ± 5.35, and for ensuring health while working, the score was 15.12 ± 3.11 (Specific details in S1 File).

### Factors associated with knowledge, attitude and practice scores

Table 3 presents the results of the multivariate Tobit regression analysis for factors associated with knowledge scores related to occupational accident prevention among rice farmers. Farmers who participated in other occupations had significantly higher knowledge scores (Coef. = 1.307, 95% CI: 0.744 to 1.871, p < 0.001). Longer experience in rice farming was associated with a slight but statistically significant decrease in knowledge (Coef. = −0.024, 95% CI: −0.048 to −0.0002, p < 0.05). Those who had experienced an occupational accident in the past six months had lower knowledge scores (Coef. = −3.187, 95% CI: −3.900 to −2.474). Additionally, attitude scores (Coef. = 0.249, p < 0.001) and practice scores (Coef. = 0.067, p < 0.001) were positively associated with knowledge scores.

**Table 3:** Multivariate Tobit regression for identifying factors associated with knowledge score.

Variable	Coef.	95% CI (Lower)	95% CI (Upper)
Gender			
Male (ref)	–	–	–
Female	−0.181	−0.715	0.353
Age Group			
< 60 years (ref)	–	–	–
≥ 60 years	0.502	−0.141	1.145
Education			
≤ Secondary school (ref)	–	–	–
≥ High school	0.195	−0.480	0.870
Other Occupation			
No (ref)	–	–	–
Yes	1.307***	0.744	1.871
Years in Rice Production	−0.024*	−0.048	−0.0002
Labor Hours per Day	0.042	−0.081	0.165
Occupational Accident (6 months)			
No (ref)	–	–	–
Yes	−3.187	−3.900	−2.474
Attitude score	0.249***	0.187	0.312
Total practice score	0.067***	0.043	0.091

*Ref = Reference group;* p < 0.05 = *, p < 0.01 = **, p < 0.001 = ***

Table 4 shows the multivariate Tobit regression results for factors associated with attitude scores. Years of experience in rice production were negatively associated with attitude scores (Coef. = −0.026, 95% CI: −0.047 to −0.005, p < 0.05). Farmers who had experienced a work-related accident in the past six months had significantly lower attitude scores (Coef. = −1.142, 95% CI: −1.800 to −0.483, p < 0.01). Higher knowledge scores were positively associated with better attitudes (Coef. = 0.236, 95% CI: 0.179 to 0.294, p < 0.001), and higher practice scores were also positively related (Coef. = 0.126, 95% CI: 0.105 to 0.146, p < 0.001).

**Table 4:** Multivariate Tobit regression for identifying factors associated with attitude score.

Variable	Coef.	95% CI (Lower)	95% CI (Upper)
Gender			
Male (ref)	–	–	–
Female	0.442	−0.031	0.914
Age Group			
< 60 years (ref)	–	–	–
≥ 60 years	0.085	−0.484	0.654
Education			
≤ Secondary school (ref)	–	–	–
≥ High school	−0.421	−1.019	0.177
Other Occupation			
No (ref)	–	–	–
Yes	0.449	−0.055	0.953
Years in Rice Production	−0.026*	−0.047	−0.005
Labor Hours per Day	0.098	−0.011	0.208
Occupational Accident (6 months)			
No (ref)	–	–	–
Yes	−1.142**	−1.800	−0.483
Knowledge Score	0.236***	0.179	0.294
**Total practice score**	0.126***	0.105	0.146

*Ref = Reference group;* p < 0.05 = *, p < 0.01 = **, p < 0.001 = ***

Table 5 summarizes the multivariate Tobit regression analysis for factors associated with practice scores. Female farmers had significantly lower practice scores compared to male farmers (Coef. = −1.659, 95% CI: −2.933 to −0.386, p < 0.05). Increased years in rice farming was associated with a decrease in practice scores (Coef. = −0.064, 95% CI: −0.120 to −0.008, p < 0.05), as was having an occupational accident in the past six months (Coef. = −2.179, 95% CI: −3.955 to −0.402, p < 0.05). Both knowledge (Coef. = 0.459, 95% CI: 0.303 to 0.615, p < 0.001) and attitude scores (Coef. = 0.907, 95% CI: 0.761 to 1.053, p < 0.001) were strongly associated with improved practice scores.

**Table 5:** Multivariate Tobit regression for identifying factors associated with practice score.

Variable	Coef.	95% CI (Lower)	95% CI (Upper)
Gender			
Male (ref)	–	–	–
Female	−1.659*	−2.933	−0.386
Age Group			
< 60 years (ref)	–	–	–
≥ 60 years	0.559	−0.975	2.094
Education			
≤ Secondary school (ref)	–	–	–
≥ High school	0.999	−0.614	2.613
Other Occupation			
No (ref)	–	–	–
Yes	−0.200	−1.560	1.160
Years in Rice Production	−0.064*	−0.120	−0.008
Labor Hours per Day	0.173	−0.122	0.468
Occupational Accident (6 months)			
No (ref)	–	–	–
Yes	−2.179*	−3.955	−0.402
Knowledge Score	0.459***	0.303	0.615
Attitude Score	0.907***	0.761	1.053

*Ref = Reference group;* p < 0.05 = *, p < 0.01 = **, p < 0.001 = ***

## Discussion

### Summary of main findings

This study provides a comprehensive analysis of occupational accident prevention among rice farmers in Thai Binh province, Vietnam. Among the 1,171 participants, 17.5% reported experiencing at least one occupational accident in the past six months. Overall, the results indicated moderate levels of KAPrelated to occupational safety. Multivariate analyses revealed that occupational accident history, years of farming experience, and participation in other occupations were significantly associated with knowledge, attitudes, and safety practices.

### Knowledge

Occupational safety and health are best evaluated through the identification and mitigation of workplace hazards [26]. In this study, farmers displayed the highest knowledge in preventing injuries related to physical impact (mean: 3.90/5) and chemical hazards from plant protection chemicals (PPC) (mean: 3.85/5), consistent with findings from Baksh et al. (2015) [27], who noted that farmers tend to be more aware of chemical risks than physical ones. Likewise, Mirakzadeh et al. (2018) reported high levels of pesticide safety knowledge in Iranian farmers, particularly in relation to spraying techniques [28].

However, knowledge about ergonomic hazards was notably low (mean: 1.52/3), mirroring previous research that highlighted a widespread lack of awareness about musculoskeletal and posture-related risks [29–31]. This suggests that ergonomic safety—though equally critical—is often overlooked in farmer training programs, likely due to its less immediate visibility and delayed onset of symptoms.

### Attitudes

The mean attitude score (34.47/45) indicated generally positive safety beliefs among rice farmers. Most farmers strongly valued personal safety, especially in terms of recognizing occupational accidents as a serious issue and affirming the preventability of such events. This aligns with studies such as Rini & Aswin (2023) [32], which found that farmers expressed favorable attitudes toward labor protection. However, the lowest scoring item was the perceived importance of seeking immediate medical care following an accident (mean: 3.62), suggesting that despite general awareness, emergency response behaviors may be neglected. This observation is supported by literature showing mixed attitudes toward occupational health compliance among farmers, often due to limited information or ingrained fatalism [33,34]. Without consistent reinforcement of the importance of post-injury care, even well-informed individuals may not prioritize immediate medical action.

### Practices

Despite moderate knowledge and relatively positive attitudes, actual safety practices among rice farmers remained limited, with an average practice score of 63.63 out of 85 points (equivalent to 23.24 out of 30). While there is no established threshold for interpreting this score, the result suggests that farmers implemented only a portion of recommended safety behaviors in their daily work. This gap between awareness and action is consistent with international findings. For example, studies from Ethiopia reported that 37.7–42% of farmers never used personal protective equipment (PPE) [35,36], a pattern also observed in our data. Farmers in this study were more likely to use protective gear during pesticide application than during other routine tasks, such as lifting or operating machinery.

The persistence of this behavioral gap has been widely documented. Dita et al. (2019) emphasized that knowledge is a necessary but not sufficient condition for safe behavior [37]. Our findings showed significant correlations among the knowledge, attitude, and practice domains, in line with Gustina et al. (2019) [38] and Bryan et al. (2022) [39], who found strong associations between higher knowledge levels and reduced accident risk. Similarly, Nurul et al. (2022) reported that positive attitudes were critical for consistent PPE use [40].

### Factors associated with knowledge, attituded and practices

Participation in other occupations was positively associated of knowledge, likely due to exposure to more regulated or formal work environments. This suggests that diversified work experience may enhance understanding of safety norms. Conversely, years of experience in rice farming were negatively associated with knowledge scores. Similar findings by Bryan et al. (2022) [39] and Denton (2014) [41] indicate that long-term exposure can foster complacency, as farmers may develop habitual behaviors that do not reflect updated safety standards.

Attitudes were positively correlated with the knowledge, reaffirming the role of information in shaping beliefs. However, both long-term experience and accident history were associated with more negative attitudes—likely due to normalization of risk or resignation to unsafe conditions. These findings are consistent with behavioral models where repeated exposure without adverse consequences leads to decreased perceived vulnerability.

In terms of practices, female farmers, those with more years of experience, and those with recent accidents showed poorer safety behaviors. In contrast, higher knowledge and attitude scores were positively associated with better practices, reflecting findings by Wismaningsih & Oktaviasari (2015), who highlighted the combined impact of awareness and mindset on PPE adoption [42]. The inverse relationship between accident history and safety practice may indicate a lack of post-accident intervention or insufficient training reinforcement after incidents.

### Policy and public health implications

The findings of this study highlight critical gaps in occupational safety among rice farmers in Thai Binh, with broader relevance for agricultural communities across Vietnam and other low- and middle-income countries. At the provincial level, health departments and agricultural cooperatives should collaborate to organize community-based safety training programs, including hands-on workshops on accident prevention, ergonomic practices, and emergency response. Mobile education units and peer-led safety talks conducted through farmer unions and commune health stations can improve access to knowledge, especially for older and long-term farmers who may underestimate risks or underreport injuries.

At the national level, the Ministry of Health and the Ministry of Agriculture and Rural Development should integrate occupational health and safety (OHS) modules into existing farmer extension services, with a focus on routine health screening, safe pesticide use, and injury management. The government should also promote the subsidization and wide distribution of PPE and consider mandating basic first-aid training for those engaged in agricultural labor. In addition, enhancing national surveillance systems to monitor occupational accidents in farming would provide real-time data to guide timely interventions and policy development.

Internationally, these findings support ongoing efforts by global organizations such as the International Labour Organization and the World Health Organization to improve agricultural safety through cross-country collaboration, policy harmonization, and capacity-building initiatives. Pilot interventions informed by this study could be adapted and scaled in other rice-producing countries in Southeast Asia, South Asia, and sub-Saharan Africa—where informal labor, aging farming populations, and limited OHS access remain challenges. Donor agencies and development partners are encouraged to support research-to-practice translation projects that prioritize farmer safety as a pillar of sustainable food systems.

### Limitations

This study has several limitations. First, due to its cross-sectional design, causal relationships between variables cannot be established. Second, the use of self-reported data may introduce recall bias, particularly regarding past occupational accidents, PPE use, and safety practices, as well as potential social desirability bias. Third, the study focused exclusively on rice farmers in Thai Binh province, limiting generalizability to other regions, farming systems, or crops. Fourth, although the overall response rate was high, the possibility of non-response bias cannot be entirely excluded. Finally, data collection occurred during a specific farming season, which may not capture variations in risk perception or safety behavior across different times of the agricultural calendar. Despite these limitations, the study’s robust sample size, validated and pilot-tested questionnaire, and use of multivariate modeling offer meaningful insights into occupational safety practices among Vietnamese rice farmers.

## Conclusion

This study identified significant gaps in rice farmers’ KAP related to occupational accident prevention in Thai Binh province, Vietnam. While farmers showed relatively good awareness of physical and chemical hazards, their understanding of ergonomic risks was limited, and practical implementation of safety measures remained inconsistent. Attitudes were generally positive but undervalued the importance of immediate medical care after accidents. Multivariate analysis revealed that longer farming experience and recent accident history were associated with lower KAP scores, while engagement in other occupations was associated to higher knowledge. These findings underscore the urgent need for targeted health education and integrated safety training programs to improve occupational safety practices among rice farmers, reduce injury risk, and support sustainable agricultural labor in Vietnam and comparable settings.

## Supporting information

S1 File Descriptive statistics of knowledge-attitude-practice questionnaire. (DOCX)

S2 File Survey questionnaire. (DOCX)

## References

[1] Rizka Pisceliya DM, Mindayani S. Analisis Kecelakaan Kerja Pada Pekerja Pengelasan Di CV. Cahaya Tiga Putri. Jurnal Riset Hesti Medan Akper Kesdam I/BB Medan. 2018;3(1):66. doi: 10.34008/jurhesti.v3i1.25

[2] Benjamin O. Fundamental principles of occupational health and safety. ILO. 2001;13(2):1–59.

[3] ILO. Occupational Health Services and Practice 2011. Available from: https://www.iloencyclopaedia.org/part-ii-44366/occupational-health-services/item/155-occupational-health-services-and-practice

[4] Buranatrevedh S, Sweatsriskul P. Model development for health promotion and control of agricultural occupational health hazards and accidents in Pathumthani, Thailand. Ind Health. 2005;43(4):669–76. doi: 10.2486/indhealth.43.669 16294922

[5] ILOSTAT. Informal Employment Survey 2017 inThailand. 2017.

[6] Safe Work Australia. National hazard exposure worker surveillance: Exposure to biological hazards and the provision of controls against biological hazards in Australian workplaces. 2011.

[7] Sharifirad M, Poursaeed A, Lashgarara F, Mirdamadi SM. Risk factors for musculoskeletal problems in paddy field workers in northern iran: a community-based study. J Res Med Sci. 2022;27:77. doi: 10.4103/jrms.jrms_1024_21 36438072 PMC9693728

[8] Beseler CL, Rautiainen RH. Lack of agreement between safety priorities and practices in agricultural operators: a challenge for injury prevention. Safety. 2022;8(2):39. doi: 10.3390/safety8020039

[9] Edwards JP, Kuhn-Sherlock B. Opportunities for improving the safety of dairy parlor workers. J Dairy Sci. 2021;104(1):419–30. doi: 10.3168/jds.2020-18954 33189265

[10] Kim Y, Kim D, Park K, Kim D. A study on analysis of industrial injury characteristics of aging workers in agriculture. 대한인간공학회지. 2014;33(6):477–86.

[11] Kogler R. Near accidents with agricultural vehicles, machinery and equipment in Austria in the year 2013. Agric Eng Int: CIGR J. 2015;17(1).

[12] Lekei EE, Ngowi AV, London L. Farmers’ knowledge, practices and injuries associated with pesticide exposure in rural farming villages in Tanzania. BMC Public Health. 2014;14:389. doi: 10.1186/1471-2458-14-389 24754959 PMC3999359

[13] Sapbamrer R, Thammachai A. Factors affecting use of personal protective equipment and pesticide safety practices: a systematic review. Environ Res. 2020;185:109444. doi: 10.1016/j.envres.2020.109444 32247154

[14] Keifer MC. Effectiveness of interventions in reducing pesticide overexposure and poisonings. Am J Prev Med. 2000;18(4 Suppl):80–9. doi: 10.1016/s0749-3797(00)00144-6 10793284

[15] Abou Ibrahim S, Naji R, Zeineldeen H, Ghach W. Effectiveness of pesticide labels (pictograms and color codes): a cross-sectional study of farmers’ understanding and practices in Lebanon. Hum Ecol Risk Assess: Int J. 2023;29(9–10):1336–51. doi: 10.1080/10807039.2023.2266036

[16] Maddah D, Ghach W, Abi Farraj N, Yehya M, Al Khatib J, Alami NH. The first community-based intervention to promote safe pesticide use by developing knowledge, attitudes, and practices among Lebanese farmers. Hum Ecol Risk Assess: Int J. 2019;26(10):2824–35. doi: 10.1080/10807039.2019.1688639

[17] Dugger-Webster A, LePrevost CE. Following pesticide labels: A continued journey toward user comprehension and safe use. Curr Opin Environ Sci Health. 2018;4:19–26. doi: 10.1016/j.coesh.2018.03.004

[18] Demirbaş N, Cukur F, Yildiz Ö, Gölge E. Level of Knowledge, Practices and Attitudes of Dairy Farmers Regarding Food Safety in Turkey. New Medit. 2009;8.

[19] Gia NT, Khoa THA, Giang MN, Dũng NĐ, Dương LĐ, Thắng TB, et al. Thực trạng tai nạn thương tích trong lao động nông nghiệp của nông dân trồng lúa tại huyện Phú Vang, tỉnh Thừa Thiên Huế. Tạp chí Y học Dự phòng. 2024;33(6 Phụ bản):338–45. doi: 10.51403/0868-2836/2023/1436

[20] Kawakami T, Van VN, Theu NV, Khai TT, Kogi K. Participatory support to farmers in improving safety and health at work: building WIND farmer volunteer networks in Viet Nam. Ind Health. 2008;46(5):455–62. doi: 10.2486/indhealth.46.455 18840935

[21] Nguyen MH, Van Ngo T, Tran AQ, Vu LG, Phan NT, Pham ST, et al. Occupational accidents among rice farmers in Northern Vietnam. Sci Rep. 2024;14(1):27243. doi: 10.1038/s41598-024-78443-x 39516541 PMC11549280

[22] Phung DT, Connell D, Miller G, Rutherford S, Chu C. Needs assessment for reducing pesticide risk: a case study with farmers in Vietnam. J Agromedicine. 2013;18(4):293–303. doi: 10.1080/1059924X.2013.826605 24125044

[23] Ngân H. Sản xuất lúa, gạo theo hướng bền vững. Báo Thái Bình. 2024.

[24] Organization WH. Public health impact of pesticides used in agriculture. Public health impact of pesticides used in agriculture. 1990. pp. 128.

[25] International Labour Office. Safety and Health in Agriculture. ILO Code of Practice. Geneva (CHE): International Labour Office; 2011.

[26] Idirimanna IASD, Jayawardena LNAC. Factors affecting the health and safety behavior of factory workers. 2011.

[27] Kurina B, Wayne G, Lendel N. Farmers knowledge, attitudes and perceptions of occupational health and safety hazards in Trinidad, West Indies and implications for the agriculture sector. J Agric Ext Rural Dev. 2015;7(7):221–8. doi: 10.5897/jaerd2015.0672

[28] Moradhaseli S, Mirakzadeh AA, Rostami F, Ataei P. Assessment of the farmers’ awareness about occupational safety and health and factors affecting it; a case study in Mahidasht, Kermanshah Province. Health Educ Health Promo. 2018;6(1):23–9. doi: 10.29252/hehp.6.1.23

[29] Fathallah FA. Musculoskeletal disorders in labor-intensive agriculture. Appl Ergon. 2010;41(6):738–43. doi: 10.1016/j.apergo.2010.03.003 20398891

[30] Kirkhorn SR, Earle-Richardson G, Banks RJ. Ergonomic risks and musculoskeletal disorders in production agriculture: recommendations for effective research to practice. J Agromedicine. 2010;15(3):281–99. doi: 10.1080/1059924X.2010.488618 20665313

[31] Dianat I, Afshari D, Sarmasti N, Sangdeh MS, Azaddel R. Work posture, working conditions and musculoskeletal outcomes in agricultural workers. Int J Ind Ergon. 2020;77:102941. doi: 10.1016/j.ergon.2020.102941

[32] Willia Novita Eka R, Budi A. Palm Oil Farmers’ Perceptions on the Use of Personal Protective Equipment (PPE) in Rantau Rasau District, Tanjung Jabung District, Jambi Province. East Asian J Multidiscip Res. 2023;2(1):319–30. doi: 10.55927/eajmr.v2i1.2623

[33] Kayendeke M. Knowledge, attitude and practices among rice farmers on the use of personal protective equipment in Butebo Sub County, Butebo District. 2019.

[34] Moradhaseli S, Ataei P, Mirakzadeh A, Rostami F. Assessment of the farmers’ awareness about occupational safety and health and factors affecting it; a case study in Mahidasht, Kermanshah Province. Environmental impact assessment Recognition of Entrepreneurial Opportunities among Rural Youths View project. 2023;6.

[35] Gesesew HA, Woldemichael K, Massa D, Mwanri L. Farmers knowledge, attitudes, practices and health problems associated with pesticide use in rural irrigation villages, southwest Ethiopia. PLoS One. 2016;11(9):e0162527. doi: 10.1371/journal.pone.0162527 27622668 PMC5021266

[36] Endalew M, Gebrehiwot M, Dessie A. Pesticide Use Knowledge, attitude, practices and practices associated factors among floriculture workers in Bahirdar City, North West, Ethiopia, 2020. Environ Health Insights. 2022;16. doi: 10.1177/11786302221076250 35153486 PMC8832573

[37] Dita M, Atmojo TB, Sari Y, Susilawati TN. The correlation between knowledge about occupational accidents and safe work behaviors among employees at the production division of PT X Indonesia. KnE Life Sci. 2019;4(12):123. doi: 10.18502/kls.v4i12.4165

[38] Gustina M, Rahmawati UM, Zolendo NS. Hubungan Tingkat Pengetahuan Dan Penggunaan Alat Pelindung Diri (APD) Dengan Kejadian Gangguan Kesehatan Pada Petani Pengguna Pestisida Di Desa Simpang Pino Kecamatan Ulu Manna Tahun 2018. J Nurs Pub Health. 2019;7(1):25–9. doi: 10.37676/jnph.v7i1.758

[39] Bryan J, Ratu J, Oematan G, Umbu Roga A. Relationship between behavior and work accident in rice farmers using pesticides in Oebobo Village, Batu Putih District. Asian J Logist\ Manag. 2022;1(2):74–83. doi: 10.14710/ajlm.2022.16715

[40] Nurul H, Entianopa E, Renny L. Faktor yang berhubungan dengan perilaku penggunaan alat pelindung diri (apd) pada petani penyemprot pestisida di puskesmas paal merah II. Jurnal Inovasi Penelitian. 2022;2(9):3039–46. doi: 10.47492/jip.v2i9.1272

[41] Denton G. Determining underlying psycho-social factors influencing farmers’ risk related behaviours (both positively and negatively) in the Republic of Ireland. Dublin, Ireland: Health and Safety Authority; 2014.

[42] Wismaningsih E, Oktaviasari D. Factors related the use of personal protective equipment (PPE) in farmers in Ngantru Tulungagung District. J Wiyata. 2015;2(2):102–7.

