# Supplementary material for: Knowledge, attitudes, practices of occupational accident prevention and related factors among rice farmers in Northern Vietnam
Source: PLoS One. 2025 Jul 15;20(7):e0328474. doi: 10.1371/journal.pone.0328474 (PMC12262846; doi:10.1371/journal.pone.0328474)
Supplement: S1 File — (DOCX) [file pone.0328474.s001.docx]

**SUPPORTING INFORMATION**

**S1 Table. Knowledge of preventing occupational accidents of rice farmers**

| **Knowledge** | **Mean** | **SD** | |
| --- | --- | --- | --- |
| **Preventing accidents caused by equipment, machinery, and labor tools** | | | |
| Read the safety instructions carefully before operating agricultural equipment and machinery | 0.68 | | 0.47 |
| Farm equipment and machinery are regularly maintained and serviced | 0.62 | | 0.49 |
| Wear necessary protective equipment when operating machinery and using agricultural labor tools | 0.58 | | 0.49 |
| Ensure electrical safety in the work area | 0.62 | | 0.49 |
| **Preventing accidents caused by animals** | | | |
| Stay calm, move slowly, and pay attention to your surroundings when in contact with animals | 0.73 | | 0.44 |
| Do not make loud noises when in contact with animals | 0.64 | | 0.48 |
| Do not continuously hit, kick, or push animals | 0.66 | | 0.47 |
| **Preventing accidents caused by physical impact** | | | |
| Use anti-slip boots and shoes when working in the fields | 0.78 | | 0.42 |
| Wear hats and caps when working outdoors | 0.85 | | 0.36 |
| Wear long, cool clothes when working in the fields | 0.81 | | 0.39 |
| Drink enough water | 0.69 | | 0.46 |
| Do not work in the middle of the hot noon (11 am-2 pm) | 0.77 | | 0.42 |
| **Prevent accidents due to PPC** | | | |
| Use pesticides that are safe for health and have labels and expiration dates | 0.75 | | 0.44 |
| Wear protective gear when using PPC | 0.81 | | 0.39 |
| Store PPC in a separate place and have markings for use | 0.68 | | 0.47 |
| Do not talk, eat, or drink when spraying or mixing PPC | 0.80 | | 0.40 |
| Change clothes and shower after spraying or mixing PPC | 0.81 | | 0.39 |
| **Prevent accidents due to ergonomics** | | | |
| Change posture frequently when working | 0.54 | | 0.50 |
| Lift and move objects (rice, paddy, fertilizer...) with correct posture | 0.52 | | 0.50 |
| Exercise, relax muscles regularly | 0.46 | | 0.50 |

**S2 Table. Attitude of preventing occupational accidents of rice farmers**

| **Attitude** | **Mean** | **SD** |
| --- | --- | --- |
| Occupational accidents in rice production are a severe problem | 3.87 | 0.74 |
| Any farmer is at risk of occupational accidents during rice production | 3.88 | 0.76 |
| Personal safety when working in rice production is paramount | 3.93 | 0.85 |
| Occupational accidents in rice production can be prevented | 3.89 | 0.85 |
| Ensuring a safe working environment in rice production is necessary to reduce the risk of occupational accidents | 3.92 | 0.84 |
| I am willing to carry out activities to prevent accidents in rice production | 3.88 | 0.78 |
| Training to improve knowledge and practice for rice growers on occupational safety and hygiene activities is essential | 3.75 | 0.79 |
| Going to a medical facility for first aid immediately after an occupational accident is necessary | 3.62 | 0.77 |
| I am willing to remind and advise when I see other farmers not properly implementing measures to prevent occupational accidents in rice production | 3.73 | 0.79 |

**S3Table. Practice of preventing occupational accidents of rice farmers**

| **Practice** | **Mean** | **SD** |
| --- | --- | --- |
| **Safety of machinery, equipment** | | |
| Read the instructions carefully before using machinery and equipment | 3.74 | 1.01 |
| Equipment and machinery are regularly maintained and serviced | 3.58 | 1.00 |
| Power sources leading to machinery and equipment are shielded and covered | 3.77 | 1.03 |
| **Safety in using PPC** | | |
| Use plant protection chemicals that are safe for health, have full labels, and are within the expiry date | 4.05 | 0.84 |
| Do not eat, drink, or talk when mixing or spraying chemicals | 3.58 | 1.17 |
| Store plant protection chemicals in a separate place | 3.62 | 1.20 |
| Have a complete record of plant protection chemical use | 2.93 | 1.51 |
| **Occupational protection safety** | | |
| Wear a mask | 3.97 | 1.00 |
| Goggles to protect eyes | 3.62 | 1.15 |
| Protective gloves | 3.93 | 1.04 |
| Protective clothing when exposed to PPC | 3.84 | 1.12 |
| Wear protective boots | 3.92 | 1.05 |
| Wear long, airy clothes, wear a hat when working outdoors | 3.97 | 1.04 |
| **Ensure health when working** | | |
| Routine health examination | 3.27 | 1.21 |
| Exercise regularly | 3.49 | 1.18 |
| Ensure a nutritious diet and drink enough water every day. Limit the use of stimulants (alcohol, beer, etc.) | 3.92 | 0.89 |
| Clean your body after working | 4.44 | 0.76 |
