# Supplementary material for: Knowledge, attitudes, practices of occupational accident prevention and related factors among rice farmers in Northern Vietnam
Source: PLoS One. 2025 Jul 15;20(7):e0328474. doi: 10.1371/journal.pone.0328474 (PMC12262846; doi:10.1371/journal.pone.0328474)
Supplement: S2 File — (DOCX) [file pone.0328474.s002.docx]

**CURRENT SITUATION AND KNOWLEDGE, ATTITUDE, PRACTICE ON OCCUPATIONAL ACCIDENT PREVENTION OF RICE FARMER**

**I. General information**

Full Name: ……….......................................................................................................................
Address: …………………………………………………………………………………
Phone Number: …………………………………………………………………………………

| Gender | 1. Male  2. Female |
| --- | --- |
| Year of Birth / Age (Gregorian calendar) | Year of Birth: ...............  Age: .................. |
| Highest Educational Attainment | 1. Illiterate  2. Primary School  3. Secondary School  4. High School  5. Vocational/College  6. University/Postgraduate |
| Current Marital Status | 1. Single  2. Married  3. Separated/Divorced  4. Widowed  5. Other (Specify): ............... |
| How many people live in your household | ............... persons |
| Who is the main income earner in your household | 1. Yourself  2. Wife/ Husband  3. Other relatives |
| Household economic status | 1. Poor  2. Near poor  3. Not classified in the above categories |

**II. Lifestyle Information**

| Do you have the habit of drinking alcohol or beer? | 0. No  1. Quit  2. Yes |
| --- | --- |
| If YES, how often do you drink recently? | 1. Daily  2. Weekly (1–4 times/week)  3. Monthly (1–4 times/month)  4. Other (Specify): .................... |
| Average amount per drinking session | ………… ml (beer)  ………… ml (liquor) |
| Do you smoke cigarettes? | 0. No  1. Quit  2. Yes |
| If YES, how many cigarettes do you smoke per day? | 1. ≤10 cigarettes/day  2. 11–20 cigarettes/day  3. 21–30 cigarettes/day  4. 31 cigarettes/day  5. Don’t remember |
| Do you exercise daily? | 0. No  1. Yes |
| If YES, how many minutes do you exercise on average per day? | .................. minutes |

### III. Agricultural Labor Information

| **Total time you have worked in the rice production to date** | ……… years |
| --- | --- |
| **In the past 6 months, average time per day to perform rice production activities** | ……….hours |

**IV. Current situation of occupational accidents of rice farmers**

**TN1.** In the past 6 months, have you ever had an occupational accident while performing rice production activities?

1. Yes
2. No → Skip to KAP
3. Don't remember / No answer → Skip to KAP

**TN2. If yes, n**umber of occupational accidents in the past 6 months?

…………………… times

**TN3:** Cause of the occupational accident?

1. Fall

2. Lifting/carrying

3. Animal-related (bite, butt...)

4. Machinery accident

5. Sharp objects

6. Traffic accident

7. Heatstroke/sunstroke

8. Pesticide poisoning

9. Electric shock

10. Other (Specify): ..........

**V. KNOWLEDGE, ATTITUDE, PRACTICE OF PREVENTING OCCUPATIONAL ACCIDENTS OF RICE FARMERS**

**I. Knowledge**

Each of the following statements represents your opinion and knowledge about measures to prevent occupational accidents in rice production. There are 2 levels that can be displayed as follows (Circle each option):

0. Don't know 1. Know

|  | **Knowledge** | **Select** | |
| --- | --- | --- | --- |
| **Preventing accidents caused by equipment, machinery, and labor tools** | | | |
|  | Read the safety instructions carefully before operating agricultural equipment and machinery | 0 | 1 |
|  | Farm equipment and machinery are regularly maintained and serviced | 0 | 1 |
|  | Wear necessary protective equipment when operating machinery and using agricultural labor tools | 0 | 1 |
|  | Ensure electrical safety in the work area | 0 | 1 |
| **Preventing accidents caused by animals** | | | |
|  | Stay calm, move slowly, and pay attention to your surroundings when in contact with animals | 0 | 1 |
|  | Do not make loud noises when in contact with animals | 0 | 1 |
|  | Do not continuously hit, kick, or push animals | 0 | 1 |
| **Preventing accidents caused by physical impact** | | | |
|  | Use anti-slip boots and shoes when working in the fields | 0 | 1 |
|  | Wear hats and caps when working outdoors | 0 | 1 |
|  | Wear long, cool clothes when working in the fields | 0 | 1 |
|  | Drink enough water | 0 | 1 |
|  | Do not work in the middle of the hot noon (11 am-2 pm) | 0 | 1 |
| **Prevent accidents due to PPC** | | | |
|  | Use pesticides that are safe for health and have labels and expiration dates | 0 | 1 |
|  | Wear protective gear when using PPC | 0 | 1 |
|  | Store PPC in a separate place and have markings for use | 0 | 1 |
|  | Do not talk, eat, or drink when spraying or mixing PPC | 0 | 1 |
|  | Change clothes and shower after spraying or mixing PPC | 0 | 1 |
| **Prevent accidents due to ergonomics** | | | |
|  | Change posture frequently when working | 0 | 1 |
|  | Lift and move objects (rice, paddy, fertilizer...) with correct posture | 0 | 1 |
|  | Exercise, relax muscles regularly | 0 | 1 |

**II. Attitude**

Each of the following statements is your opinion and attitude towards occupational accident prevention in agricultural production. There are 5 levels of expression as follows (Circle the choice):

1: Completely disagree 2: Disagree 3: Neutral

4: Agree 5: Completely agree

|  | **Attitude** | **Select** | | | | |
| --- | --- | --- | --- | --- | --- | --- |
|  | Occupational accidents in rice production are a severe problem | 1 | 2 | 3 | 4 | 5 |
|  | Any farmer is at risk of occupational accidents during rice production | 1 | 2 | 3 | 4 | 5 |
|  | Personal safety when working in rice production is paramount | 1 | 2 | 3 | 4 | 5 |
|  | Occupational accidents in rice production can be prevented | 1 | 2 | 3 | 4 | 5 |
|  | Ensuring a safe working environment in rice production is necessary to reduce the risk of occupational accidents | 1 | 2 | 3 | 4 | 5 |
|  | I am willing to carry out activities to prevent accidents in rice production | 1 | 2 | 3 | 4 | 5 |
|  | Training to improve knowledge and practice for rice growers on occupational safety and hygiene activities is essential | 1 | 2 | 3 | 4 | 5 |
|  | Going to a medical facility for first aid immediately after an occupational accident is necessary | 1 | 2 | 3 | 4 | 5 |
|  | I am willing to remind and advise when I see other farmers not properly implementing measures to prevent occupational accidents in rice production | 1 | 2 | 3 | 4 | 5 |

**III. Practice**

Each statement below is your practice of preventing occupational accidents in the production process. There are 5 levels of expression as follows (Circle the appropriate option):

1: Never 2: Rarely 3: Sometimes

4: Often 5: Always

|  | **Practice** | **Select** | | | | |
| --- | --- | --- | --- | --- | --- | --- |
| **Safety of machinery, equipment** | | | | | | |
|  | Read the instructions carefully before using machinery and equipment | 1 | 2 | 3 | 4 | 5 |
|  | Equipment and machinery are regularly maintained and serviced | 1 | 2 | 3 | 4 | 5 |
|  | Power sources leading to machinery and equipment are shielded and covered | 1 | 2 | 3 | 4 | 5 |
| **Safety in using PPC** | | | | | | |
|  | Use plant protection chemicals that are safe for health, have full labels, and are within the expiry date | 1 | 2 | 3 | 4 | 5 |
|  | Do not eat, drink, or talk when mixing or spraying chemicals | 1 | 2 | 3 | 4 | 5 |
|  | Store plant protection chemicals in a separate place | 1 | 2 | 3 | 4 | 5 |
|  | Have a complete record of plant protection chemical use | 1 | 2 | 3 | 4 | 5 |
| **Occupational protection safety** | | | | | | |
|  | Wear a mask | 1 | 2 | 3 | 4 | 5 |
|  | Goggles to protect eyes | 1 | 2 | 3 | 4 | 5 |
|  | Protective gloves | 1 | 2 | 3 | 4 | 5 |
|  | Protective clothing when exposed to PPC | 1 | 2 | 3 | 4 | 5 |
|  | Wear protective boots | 1 | 2 | 3 | 4 | 5 |
|  | Wear long, airy clothes, wear a hat when working outdoors | 1 | 2 | 3 | 4 | 5 |
| **Ensure health when working** | | | | | | |
|  | Routine health examination | 1 | 2 | 3 | 4 | 5 |
|  | Exercise regularly | 1 | 2 | 3 | 4 | 5 |
|  | Ensure a nutritious diet and drink enough water every day. Limit the use of stimulants (alcohol, beer, etc.) | 1 | 2 | 3 | 4 | 5 |
|  | Clean your body after working | 1 | 2 | 3 | 4 | 5 |
